# Experiences of aftermath of COVID-19 in relation to social, financial and health related aspects among previously hospitalized patients: a qualitative exploration

**DOI:** 10.3389/fpubh.2023.1196810

**Published:** 2023-06-15

**Authors:** Ahmed Jojan Nandonik, Shangjucta Das Pooja, Tanvir Ahmed, Anwar Parvez, Zarina Nahar Kabir

**Affiliations:** ^1^SAJIDA Foundation, Dhaka, Bangladesh; ^2^Karolinska Institute, Huddinge, Sweden

**Keywords:** COVID-19, content analysis, qualitative research, Bangladesh, post-COVID experiences, discharged patients

## Abstract

**Background:**

There is increasing evidence of long-term consequences of COVID-19. The world has seen multidimensional impact of the pandemic and Bangladesh is no exception to that. Policymakers in Bangladesh laid out strategies to curb the initial spread of COVID-19. However, long-term consequences of COVID-19 received little or no attention in the country. Evidence suggests that people presumed to be recovered face multidimensional post-covid consequences. This study aimed to describe the aftermath of COVID-19 in relation to social, financial and health related aspects among previously hospitalized patients.

**Methods:**

This descriptive qualitative study includes participants (*n* = 14) who were previously hospitalized for COVID-19 and returned home after recovery. The participants were part of a mixed method study from which they were purposively selected. Semi-structured in-depth interviews were conducted over telephone. Inductive content analysis was used to analyze the data.

**Results:**

Twelve sub-categories emerged from the data analysis which converged into five main categories. The main categories included *perspective on physical health*, *financial struggle*, *life adjustment*, *interplay between different domains*, and *spontaneous support*.

**Conclusion:**

The lived experiences of COVID-19 recovered patients highlighted multidimensional impact on their daily lives. Physical and psychological wellbeing found to be related to the effort of restoring financial status. People’s perception about life altered due to pandemic, for few the pandemic was an opportunity to grow while others found it difficult to accept the hardship. Such multidimensional post COVID-19 impact on people’s lives and wellbeing holds considerable implication for response and mitigation plan for future related pandemics.

## Background

The impact of Coronavirus disease 2019 (COVID-19) pandemic has resulted in multidimensional challenges across the globe (1, 2). Much research has been conducted on opportunities and challenges in low-and middle-income countries (LMICs) during the pandemic (3) and risk factors and adverse outcomes among patients with COVID-19 (4, 5). Furthermore, there are evidence of population health and economic security related challenges (6, 7). There is also, documentation on impact of preventive measures including vaccination (8, 9), easing restrictions and consequences in relation to local, national and regional level strategies and policies (10, 11). In addition to health and related system challenges, COVID-19 pandemic has shown how a range of sociodemographic and economic factors can affect people’s wellbeing, i.e., due to losing employment (12), and loss of household income as a result of restriction measures like lockdown (13, 14). While, many could not go to the workplace and lost their jobs, some of the fortunate ones had the opportunity to work from home (15–17). Furthermore, social isolation (18), stigma associated with being COVID-19 positive (19), and gender-based violence due to economic insecurity, alcohol consumption and being forced to stay at home (20) were observed during the pandemic. While much of this refers to the sufferings during the active wave(s) of the pandemic, there is also evidence of physical (21, 22) and mental health (23, 24) related post COVID-19 consequences. It is important to note that, while much of these evidence documents health (including system) related impact, there is also evidence of non-health related impact due to the COVID-19 pandemic across the globe (25).

The non-health impact of the COVID-19 pandemic can be observed at individual, household, and community levels as social and economic disruption and can vary in intensity (26, 27). State support (health and non-health) was available in high-income countries (HICs) to tackle these impacts during the pandemic. For example, the Furlough scheme was introduced in the UK where companies allowed employees to take temporary leave from work (28). Similar schemes and financial support were also introduced in other HICs including United States of America, Ireland, Sweden, Italy and Germany (29). Unlike HICs, formal health care and social support mechanisms are very limited in low and middle-income countries (LMICs) (30). As a result, families have been reported to have primarily rely on informal care or community support network when it comes to caregiving in case of illness, even emotional or financial support when in need (30). HICs like the USA (United States of America), Canada, Australia and a few others implemented moratorium on evictions, mortgage payments and other measures to ensure citizens do not lose housing during the pandemic (31–33). Also, remote learning and remote working initiatives were introduced (34). On the other hand, LMICs executed lockdown measures to tackle the spread of COVID-19. Additionally, limited non-health measures were taken to mitigate social, economic and psychological impacts of the pandemic. When state support had been limited, many communities came together to provide support and aid to those who affected by the pandemic (35). The robustness of these community efforts are not widely known and the impact of such effort is not well documented. There is a dearth of literature that discusses the interplay of health, social and financial aspects after the initial hit of COVID-19 pandemic and how such multidimensional impact affected one’s life after COVID-19.

The experience of the COVID-19 pandemic continues to be a learning curve for policymakers in the public health sector. The multi-dimensional impact of the pandemic calls for attention particularly in resource constrained countries such as Bangladesh where support from the State at times of crisis is limited. Hence, it is important to understand how the experience of COVID-19 has affected everyday lives of individuals even after recovery from its acute stage in order to explore possible support strategies within the family, community and by policymakers.

The objective of the study was to describe the experience of the aftermath of COVID-19 of previously hospitalized patients in relation to social, financial and health related aspects.

## Materials and methods

### Design

This descriptive qualitative study used content analysis method with inductive approach.

### Study setting and participants

The context of the study was a hospital dedicated solely to patients with COVID-19 in Bangladesh. It was set up by a non-profit organization and located in Narayanganj district, about 18 km from the capital city Dhaka. From April to December 2020, 1,022 patients were admitted to this hospital with symptoms similar to COVID-19. Of 1,022, 52 were deceased and 28 referred to other facilities. The rest of the 942 were considered for a telephone survey and 420 were reached (36). Among them, 32 (21 men and 11 women) were purposively identified and invited to participate in this qualitative study. Fourteen agreed to participate.

Of the study participants three were women and 11 men. Median age of the study participants was 44 years. Most of the participants (*n* = 12) had at least 10 years of education and lived in a joint family (*n* = 9). Majority (*n* = 10) were in paid employment.

### Data collection: tools and techniques

The data collection of this study was conducted from October 5 to November 30, 2021using semi-structured interviews. An open-ended interview guide was developed which covered the following topics in relation to experience of COVID-19: social and financial challenges, changes in daily lives after recovery, health related information and accessibility, availability and affordability of the healthcare service availed. To ensure physical distancing and infection prevention measures, the guideline was pretested with participants not included in the study but with similar sociodemographic profile to the study participants to make it compatible for telephone interviews. Prior appointment was made for each interview session which was about 45 min long on average and recorded. If more time was needed, the interview was completed in more than one session at a time preferred by the participant. Interviews were conducted by three of the co-authors (AN, SD & AP) who had previous experience in conducting qualitative interviews. At least three phone calls were made at different times before considering a potential participant non-responsive.

### Ethical considerations

This study was conducted with ethical approval obtained from Bangladesh Medical Research Council (BMRC); reference number 315115102020. All interviews were conducted after obtaining informed consent from each participant informing them of the study objective, voluntary nature of their participation in the study and right to withdraw without any consequences. The interviews were recorded and stored on a secured server with IDs to anonymize the participants. These records were accessible to the research team only.

### Data analysis

The interviews were transcribed verbatim in Bangla. Content analysis with inductive approach (37) was used for data analysis. The co-authors read the transcripts multiple times. AN and SD read all the transcript and ZK, TA and AP read a selected few to encourage triangulation in the data analysis. Each author coded each transcript independently and identified subcategories. All codes were later reorganized into subcategories and then into categories by the researchers in an iterative process through regular team meetings. Key quotes presented in the results section were translated into English by AN and SD. Quotes by participants are referred to as “P” followed by participant’s number (Table 1).

**Table 1:** Overview of categories and subcategories that emerged from data analysis.

Category	Subcategory
Perspective on physical health	Experience of new symptoms
	Positive behavioral change
Financial struggle	Financial impact
	Financial adjustment
	Financial burden of continuing treatment
Life adjustment	Social preference
	Change in regular activities
Interplay between different domains	Overlapping of physical and financial concerns with mental health concerns
	State of mind
Spontaneous support	Immediate support from family helped in physical recovery
	Got more than expected: support from neighbors, relatives and friends
	Support from workplace

## Results

The analysis revealed five key categories: perspective on health, financial struggle, life adjustment, interplay between different domains and spontaneous support. These five categories explore the lived experience of previously hospitalized participants who recovered from COVID-19 and continued with their daily routine.

### Perspective on physical health

Participants narrated variations in their health trajectories after recovering from COVID-19, which further manifested behavioral changes in their lifestyles and related adjustments. Most of them reported experiencing new symptoms and declining physical health.

#### Experience of new symptoms

Participants described that recovering from COVID-19 was a unique experience. Some of the post COVID-19 consequences were exhaustion and/or breathlessness when performing everyday activities, including climbing stairs. “*Feeling tired even after having rested*” was often mentioned by the participants including difficulty in waking up early in the morning. Participants referred to their experience as physical “weakness” in general which they did not experience before suffering from COVID-19. As some participants stated:

“I often get sick or I feel tired, like while doing heavy work my body becomes weak”. (P1)

“Earlier I could lie down and sleep. Now I don’t have sound sleep, like ‘complete sleep’. Weakness is still there”. (P6)

The participants believed that they developed a number of health problems as a consequence of COVID-19. These included joint pain, body ache, shivering, slow healing of scars, intolerance to heat, and calcium deficiency. Although the respondents mentioned these as post COVID-19 experiences, none consulted with any physician. One participant suspected that the congenital anomaly of his unborn child was an aftermath of the mother’s COVID-19 infection during pregnancy. Later on, they decided not to bring that child into the world.

“…After COVID we conceived a child … through ultrasonogram we came to know that the child’s hands were small and head was very large. The doctor said that it won’t be a healthy baby … after a lot of difficult discussion the child was aborted; we felt very bad … God knows if it happened because of COVID”. (P5)

#### Positive behavioral change

Participants reported change(s) in lifestyle after they were released from the hospital. To many, experience of COVID-19 led to a growing sense of responsibility toward self-care. Eating healthy food, improved sleeping habits and hygiene practices were some of the positive behavioral changes described by the participants.

“I have managed to lose some weight and have changed my food habit. After consulting with doctor, I now go to sleep early after having dinner. Before, I used to go to sleep around 12 am or later. Now, by 10 to 10:30 pm, I go to sleep and wake up early in the morning, at the time of morning prayer”. (P6)

### Financial struggle

Almost all the participants mentioned financial struggle brought onto them due to COVID-19. The participants mentioned facing financial strains due to disruption in daily routine due to lockdown and not being able to participate in income generating activities. In addition to this, existing financial stress, costs of follow up after COVID-19 with physicians were added dimensions to the participants’ financial struggle.

#### Financial impact

COVID-19 pandemic exacerbated the economic hardship of the participants and their families. Participants running their own business struggled to maintain day-to-day operations. Some had to shut down their businesses due to non-recovery from financial loss. Such losses caused reductions in revenues and many went into debt. On top of that, price-hike due to the pandemic forced many participants to cut down on their daily expenses. A participant mentioned that his standard of living has fallen compared to what it used to be even after the situation of COVID-19 pandemic had relatively stabilized. As one of the participants mentioned:

“I have three children and I have to bear their tuition cost. It [*bearing tuition cost*] is getting difficult. I barely can cover the monthly cost, managing everything from here and there.” (P11)

Another participant stated:

“I do small businesses now which I used to do at a larger scale. Pandemic has changed the situation, made the business upside down. I’m unable to repay loan due to closure of business during the pandemic. Facing humiliation from lenders”. (P3)

#### Financial adjustment

The financial shock due to the pandemic pushed participants to make some unwanted and tough decisions. One of the participants mentioned moving further from the workplace as the previous residence became unaffordable. Another participant stopped attending social events to cut down expenses. Furthermore, regular follow up with doctors for physical ailments and related medicines took a financial toll especially on participants with limited earnings.

“Earlier I had some income through private tutoring … which has now reduced to fifty percent. Since the rent is higher, I had to move [*for cheaper residents*] although the new area is further from my workplace. It is tiring to travel but still I have to”. (P11)

#### Financial burden of continuing treatment

Many participants were required to follow up with physicians on their health status after recovering from COVID-19. This created extra financial burden for them. Some participants skipped follow-up consultations with physicians as they could not afford it. Their financial crisis during the COVID-19 pandemic resulted in this unaffordability.

“Seeing doctor means additional expense of 5-10 thousand. We don’t have the solvency to bear the expenses of those multiple recommended tests [*given by the doctor*]. This is why we refrain from going for health examination”. (P3)

### Life adjustments

Most of the participants mentioned that recovery from COVID-19 had been challenging and their life-style had changed in many ways. For some, the change was sudden and could cope with it by changing their usual lifestyle such as social interaction and daily habits.

#### Social preference

Participants talked about changes in their social interactions. The charm of festivals and family gatherings, which is an integral part of the Bengali culture, somewhat faded away. One of the participants mentioned, “*joy of shopping* [for festivals] *is not there anymore*.” Instead, she prefers being alone at home nowadays. Those who tried to return to previous ways of socialization by visiting relatives felt hesitant as they realized others were no longer open to receiving guests and preferred to maintain distance due to fear of contracting the virus. Such dilemma led people to change their “pre-COVID” social habits and the participants tended to distance themselves from social activities. One of the participants stated:

“The restrictions were actually very normal. Say my childhood friend, my nephew, I adore him a lot. I told him, if you allow, can I meet you? It’s been long since we met. But they didn't give permission. It seemed that they were afraid of catching COVID-19. I felt this way, but cannot blame them either”. (P13)

#### Change in regular activities

Considerable decrease in ability to perform regular activities due to loss of physical strength since recovery from COVID-19 was reported by few. They mentioned such debilitating state made it difficult for them to go back to their daily routines. A participant described that he lost confidence in performing heavy work which required significant physical strength:

“The way I used to perform physical chores with great strength before, can’t do it now. I become breathless, tired even with small amount of activity. Also, after a night’s sleep, I can barely wake up during the Fajr [*morning*] prayer”. (P14)

Another participant stated:

“I can’t work in the office as if my brain can’t take the workload. But before I could take any stress, I could deal with people, could talk to the factory manager. But nowadays my younger brother deals with everything on my behalf. If I anyone gives me stress, I lose my mind.”(P5)

### Interplay between different domains

Experience of changes in health (physical and mental) and financial status were not separate experiences but intertwined; one affected the other. Physical and financial concerns triggered negative emotions which led to a deterioration of well-being. This section describes the inter-connections between physical and financial concerns with mental health concern of the participants.

#### State of mind

Participants talked about experiencing a state of mind that they never experienced before. According to them, such ‘feelings’ started appearing after having suffered from COVID-19 and these were unfamiliar to them. Some of these concerns included self-doubt and memory loss. Some attributed such state of mind to financial crisis post-COVID. One participant mentioned, inactivity for a long time due to illness led to a feeling of being useless:

“If I stayed around the house till 11 in the morning, I used to feel useless”. (P 14)

Others mentioned experiencing anger and stress after suffering from COVID-19. Experience of ‘not being able to control anger’ and ‘ill temper’ in particular was reported by almost all the participants. When asked, most of the participants mentioned “*fear of being reinfected with COVID-19*” was one of the main reasons of their ill-temper and stress. According to one of the participants:

“These days, I get angrier than before. Although I try to control it, my temper flares up”. (P1)

Some participants felt sadness and depressed after suffering from COVID-19. One such participant mentioned that “*cheer*” disappeared from his life:

“I don’t feel like going out now. I used to feel cheerful before Corona and used to go out to different places, but not anymore”. (P7)

It appeared that having suffered from Covid-19 impelled few participants to take on a new perspective on life. It led them to believe that life was fragile so one should devote oneself to religion rather than rushing toward worldly matters.

“Once I had COVID-19, as Muslim my religious belief grew stronger. The world is temporary. I skipped prayer a lot and used to run after money. Now I believe this temporary world is insignificant.” (P14)

#### Overlapping of physical and financial concerns with mental health concerns

Participants described anxiety led to inadequate sleep which then led to fatigue. They reported changes in their sleep patterns ever since they had COVID-19. For some, it was difficult to fall asleep. For others, lack of sleep became a regular phenomenon. One participant stated having frequent sleep paralysis which he had never experienced before getting COVID-19. Participants described:

“I can’t sleep at all since COVID-19, maybe those tensions [*financial stress*] kept me from sleeping. Then, my body starts shivering, I feel restless. Also, I noticed one thing that when I try to sleep my legs become numb. I think I should consult with a doctor.” (P11)

“…When scarcity knocks at your door, it is difficult to control your mood…when you don’t have money it creates a lot of pressure around you from the family…” (P1)

Some participants described losing the capacity to think rationally and reduced ability to handle stress which made them feel anxious and led to deteriorated physical condition.

“I think I am more troubled by my mental health condition than physical health. Frankly speaking, my business was dependent on mental ability to work than physical labor. I was in shock thinking about how to run my business, what will I do next, from where will I get things etc. When think about it know I feel a tightness in my chest.” (P14)

Almost all the participants complained about forgetfulness after suffering from COVID-19, in particular forgetting things that happened in the recent past. This include forgetting about an incident and not being able to remember conversations that took place few days earlier. A participant thought that such problems could be due to difficulties of being attentive:

“I think I tend to forget things now. Like, I did something few days back but now I don’t remember anything about it. This happens to me”. (P2)

Many participants struggled financially during the pandemic which led to psychological distress. Loss of income and fear of losing employment, uncertainty about employees’ payments due to loss in business and being threatened with lawsuits etc. affected participants’ mental health.

“Being tense causing reduction in sleep time, I believe financial stress is the reason behind it as business was going well before [*the pandemic*]. They [*creditors*] calls my wife if my phone is switched off; if her phone is also switched off, they call our relatives. It is creating so much pressure on us, it is unbearable. This is causing turmoil in the family. We are not able to repay the loan…they [creditors] have threatened to take legal action against us…” (P3)

### Spontaneous support

One of the major factors that helped the participants during their recovery was mentioned to be moral and mental support from one’s surroundings. This could come either from the personal space or professional sphere.

#### Immediate support from family helped In physical recovery

Close family members played a crucial role when participants were affected by COVID-19. This support ranged from communicating with doctors for treatment to staying in the hospital with the participants to give them mental strength. To some, this helped in their physical recovery from COVID-19. One of the participants stated:

“I could not do any work then. My wife handled everything. She provided all the support that I needed. She stayed at the hospital with me although she was not COVID-19 positive. She was there as long as I was there. You will be surprised to know that within a month I gained the weight that I lost [*because of the care my wife took of me*]”. (P2)

#### Got more than expected: support from neighbors, relatives and friends

Support from the social sphere (other than immediate family) was mentioned to be one of the factors that aided in recovery of the participants. In spite of the fear of contracting COVID-19 among the general population, the participants expressed their gratitude toward their neighbors, relatives and friends for providing help, support and care. One of the participants stated that his neighbors helped him with daily groceries when he and his family members were sick with COVID-19. Others too mentioned of similar experiences:

“I received lot more than I expected from my relatives. They were not reluctant or unwilling. Our family bonding is very strong”. (P2)

“Everyone used to come to see me, putting an effort to help according to their ability like doing the groceries for us. They [everyone around me] really helped us when we were sick”. (P10)

#### Support from workplace

Support from the workplace, such as, ensuring job security, providing financial support, and moral support from colleagues were very much appreciated by the participants. One of the participants described that he was worried that he might lose his job as he was away from work for a long time due to COVID-19. To his surprise, he was assured of financial support from his managing director:

“When I was admitted to the hospital, I was very stressed, thinking, will I lose my job? By the grace of the God, our managing director sir informed my colleagues to assure me that I will get all the support from my workplace and should not think about it [the job]. He [employer] also said ‘we [*office*] will bear 50% of his [*participant’s*] treatment cost’”. (P2)

Also, going back to the workplace after recovery from COVID-19 was described to be inspiring. One of the participants found that all his colleagues prayed for his well-being. He was touched by the welcome he was given by everyone at his workplace:

“It [*behavior of colleagues*] was unbelievable. The day I returned to the office, everyone came to my desk saying ‘I prayed for you’. It felt really good to hear that. I felt that God returned me to my life because of all the prayers I received”. (P1)

## Discussion

This may be one of the first studies to explore the struggles of recovery of people who suffered from COVID-19. Findings of this study indicate that participants, who were hospitalized for COVID-19, experienced considerable health and financial struggle as an aftermath of the disease. Similar physical and financial struggles have been reported in other contexts. Studies reported that people hospitalized due to COVID-19 suffered from a range of health issues that disrupted their daily lives even after being released from the hospital. This was due to the fact that health-related symptoms fluctuated unpredictably affecting general well-being (38, 39). The symptoms were sometimes non-specific, multiple or re-surfaced even after Improvement from COVID-19 (40). Furthermore, a study in Bangladesh indicated that loss of income and financial struggle during COVID-19 worsened the mental condition of adult wage earners (41).

It is important to note that people of Bangladesh too, alike in other countries, faced significant financial loss (42). Our findings suggest that going back to work or working extra hours for financial recovery affected participants’ recovery process, both physically and mentally. During the waves of the pandemic, State support was targeted only toward prioritized sectors and daily wage earners (43). Once mobility restriction was lifted, one of the major concerns for individuals was to recover from the financial loss incurred during the pandemic (44, 45). Our study also showed that people faced difficulties in returning to work. Those who suffered from COVID-19 did not have the possibility of prolonging their physical rehabilitation period before going back to work. Regular and additional health check-up recommendations put added financial burden causing more stress. There is ample research indicating the negative impact on mental health due to the effects of economic uncertainty (41, 46). Our study also shows that physical health related experience interplay with financial struggle resulting in psychological distress. A recently published article makes similar connection of interplay among physical, mental and financial (and other social) factors as precursor of post COVID consequences (25). Such interplay should be considered in mitigation plan for future similar pandemic.

The findings of our study suggest that as people continued to recover, they need to adapt their lifestyle to adjust to the challenges posed by physical and mental health related vulnerabilities, and financial struggles. Contracting COVID-19 was a scary event and altered peoples’ views toward life. At personal level, the change was mostly about self-worth, personal beliefs and sometimes questioning their identity (47). Research has shown that social isolation and inactivity can result into despair (48, 49). On the other hand, studies have reported that positive attitude toward life can help adopt a healthier lifestyle, which is a lesson learned from the pandemic (50–52). Consistent with previous work, our study also observed change in lifestyle as one of the direct outcomes of the pandemic. Altogether, it is vital to recognize vulnerabilities experienced by those who recovered from what was perceived as a life-threatening illness. This highlights the importance of incorporating post pandemic mental health support as part of the mitigation plan.

South Asian countries have familial commitment and values that are quite different from that of western countries (53). Social kinship networks (54) are believed to be embedded within the culture. A family member usually takes the role of primary caregiver and decision maker when it comes to healthcare of the sick, particularly in case of children, older persons and often adult women (55). During the COVID-19 pandemic, family caregiving remained the main source of support in LMICs. Our study has revealed that initial support for the patients with COVID-19 came from the immediate family members, which continued throughout the recovery period. Furthermore, other social networks including the patients’ workplace provided psychological and, in some cases, financial support. A study in UK similarly found that friends and family acted as informal caregivers by often adjusting their own behavior to align their activities with that of persons with long COVID (47). A study in China reported similar experience that COVID-19 brought survivors closer to their family and friends (52). However, in explaining rehabilitation needs of COVID-19 survivors a study indicated that patients sometimes tended to refrain from sharing their experience of physical and psychological symptoms to spare their family members (50, 56). Thus, culturally appropriate interventions to address future pandemics are desirable where the State can include family members in the rehabilitation process of patients.

This study reports experiences of the immediate aftermath of COVID-19 on people’s lives in Bangladesh. Future research is needed to systematically investigate the interplay between social, financial, and health-related domains affected by COVID-19 in the long term. It is important to note that the subjective experiences have not only been negative but positive too. Further research can help to identify the areas of positive experiences to strengthen those aspects when addressing future challenges.

## Limitation

It was not possible to conduct in-person interviews due to the restrictions on mobility and physical distancing in Bangladesh during the pandemic. This may have resulted in high non-response rate and a male dominant group of participants. This resulted in lack of diversity in terms of lived experience of participants, particularly women.

## Conclusion

The study provides an insight into the lived experiences of patients of COVID-19 after recovery and its multidimensional impact on their lives. Restoring financial status after the pandemic was a major stressor affecting participants’ physical and psychological wellbeing. Results indicate that the voices of long-haulers need to be heard and clarity required about Long COVID to support patients and their families in terms of knowledge about the long-term consequence of the disease and how to address them. The pandemic altered peoples’ perceptions toward life in both positive and negative ways. Some found it difficult to accept the hardship resulted by the pandemic while others adopted positive personal growth.

## Data availability statement

The raw data supporting the conclusions of this article will be made available by the authors, without undue reservation.

## Ethics statement

The studies involving human participants were reviewed and approved by Bangladesh Medical Research Council (BMRC). Written informed consent for participation was not required for this study in accordance with the national legislation and the institutional requirements.

## Author contributions

AN, SD, TA, and ZK contributed to the conception and design of the study. AP organized the database. AN and SD wrote the first draft of the manuscript. AP, TA, and ZK wrote sections of the manuscript. All authors contributed to the article and approved the submitted version.

## Conflict of interest

The authors declare that the research was conducted in the absence of any commercial or financial relationships that could be construed as a potential conflict of interest.

## Publisher’s note

All claims expressed in this article are solely those of the authors and do not necessarily represent those of their affiliated organizations, or those of the publisher, the editors and the reviewers. Any product that may be evaluated in this article, or claim that may be made by its manufacturer, is not guaranteed or endorsed by the publisher.
